# What Motor Skills Do Children Perceive as Important? A Child‐Centred Exploration Using the Motor Coordination Questionnaire

**DOI:** 10.1111/cch.70230

**Published:** 2025-12-29

**Authors:** Marisja Denysschen, Rosemary Xorlanyo Doe‐Asinyo, Dané Coetzee, Wilmarié du Plessis, Emmanuel Bonney, Bouwien Smits‐Engelsman

**Affiliations:** ^1^ Physical Activity, Sport and Recreation (PhASRec), Faculty of Health Sciences North‐West University Potchefstroom South Africa; ^2^ London School of Occupational Therapy Brunel University London Uxbridge UK; ^3^ School of Kinesiology University of Minnesota Minneapolis USA; ^4^ Masonic Institute for the Developing Brain University of Minnesota Minneapolis USA

**Keywords:** children, developmental coordination disorder, low‐resource settings, motor coordination questionnaire, motor skills, perceived competence

## Abstract

**Background:**

Understanding children's perceptions of motor skill importance is essential for designing motivating and participatory interventions. However, little is known about which motor activities children with and without motor coordination difficulties value, particularly in culturally diverse, low‐resource settings. This study explored how children perceive the importance of motor activities and how these views are shaped by cultural context, sex and motor skill level.

**Methods:**

A culturally adapted version of the motor coordination questionnaire (MoCQ) was administered to 1438 children aged 6–13 years in South Africa and Ghana. The MoCQ includes sections on perceived competence (MoCQ‐C) and perceived importance (MoCQ‐I). Motor skill level was assessed using the Movement Assessment Battery for Children, Second Edition in South Africa and the Developmental Coordination Disorder Questionnaire in Ghana. Children were classified into two motor coordination groups: as typically developing (TD) or having probable developmental coordination disorder (pDCD). Importance ratings from the MoCQ‐I were compared across countries, sex and motor coordination groups.

**Results:**

Total MoCQ‐I scores differed significantly between countries (*p* < 0.001, *d* = 0.235), sex (*p* = 0.031, *d* = 0.134) and motor coordination groups (*p* < 0.001, *d* = 0.335). Children in both countries rated self‐care, handwriting and household chores as highly important. Ghanaian children rated kicking, throwing, running, hopping and indigenous games more often as important. South African children gave a higher importance rating to handwriting. Males rated kicking, team sports and biking as more important, whereas females rated dancing higher. Differences between TD and pDCD groups at the item level were more pronounced in Ghana compared to South Africa.

**Conclusion:**

Children's perceptions of motor activity importance are shaped by cultural context, sex and motor proficiency. These findings highlight the need for culturally responsive, child‐centred interventions. While the MoCQ‐I effectively captures key activities, further expansion is recommended to enhance cultural inclusivity and relevance.

## Summary

Children in South Africa and Ghana identified self‐care, neat writing and household chores as highly important, while the perceived importance of specific motor activities varied according to cultural and environmental context.

Sex‐related differences in activity importance were evident, with boys placing greater importance on ball skills and team sports and girls valuing dancing more highly.

Children with probable developmental coordination disorder (pDCD) rated many motor activities as less important than typically developing children, suggesting that motor difficulties may influence perceived meaningfulness of participation.

Children's perceptions of important activities were shaped by sociocultural context, gender norms and motor coordination, underscoring the need for child‐centred and culturally responsive approaches in assessment, intervention and physical activity promotion.

## 1 Introduction

Understanding which motor activities children perceive as important is essential for designing interventions that are meaningful, motivating and relevant to their lives. In paediatric rehabilitation, incorporating children's preferences—that is, the activities they enjoy or feel drawn to—into goal setting is considered best practice, as it enhances engagement and supports functional outcomes (Costa et al. 2016). Yet, despite this recognition, children's voices are often underrepresented in research and clinical decision‐making. To strengthen child‐centred care, it is important to consider children's preferences (what they like to do), the perceived importance (what they consider important to be able to master) and their actual participation (what activities they do in their daily life). These constructs are related but not interchangeable: Preferences reflect affective motivation, perceived importance reflects cognitive evaluation and actual participation reflects behaviour shaped by contextual opportunities and constraints. Gathering information about perceived importance allows a better understanding of children's motor (dis)engagement.

Children's preferences for activities are shaped by a complex interplay of cultural, environmental, familial and individual influences (Brown et al. 2011; Bult et al. 2014). For example, rural Australian children engage more in recreational and self‐improvement activities like helping on farms (Brown et al. 2011), while cultural norms in Israel and South Africa influence preferences for religious, domestic or community‐centred tasks (Engel‐Yeger and Jarus 2008; Moses 2008; Visagie et al. 2017). These findings underscore the importance of considering cultural context when designing interventions, particularly in low‐resourced settings.

Parental expectations also shape children's activity choices, often reflecting beliefs about development and future success (Alexander et al. 2014). However, children's preferences may not align with their actual participation. Yalon‐Chamovitz et al. (2006) found no correlation between preferred and actual activities during school breaks, suggesting that preferences may reflect aspirations rather than routine engagement. This highlights the need to understand not only what children do but what they perceive as important and want to learn, especially when planning individualized interventions.

This becomes even more critical for children with lower motor competence, such as those with developmental coordination disorder (DCD), a neurodevelopmental condition marked by significant motor coordination difficulties (APA 2013). Children with DCD often struggle with everyday tasks and are more likely to experience reduced self‐efficacy, physical fitness and participation (Cairney et al. 2017; De Oliveira Beliche et al. 2025; Denysschen et al. 2021; Engel‐Yeger and Kasis 2010; Wright et al. 2019). Task‐oriented training, which focuses on goals meaningful to the child, has shown promise in improving outcomes (Christiansen 2018; Farhat et al. 2025; Rameckers et al. 2023; Smits‐Engelsman et al. 2013; Smits‐Engelsman et al. 2018). However, research suggests that children with DCD may avoid socially visible or challenging activities, preferring solitary or passive tasks (Engel‐Yeger and Kasis 2010; Izadi‐Najafabadi et al. 2019; Jarus et al. 2011). For instance, Jasmin et al. (2018) found that children with DCD often chose home‐based play, while Mimouni‐Bloch et al. (2016) observed a preference for sedentary activities like computer games. These patterns may reflect attempts to avoid failure or embarrassment, further limiting participation and illustrating that actual participation may reveal child constraints, not their importance rankings.

Sex differences in the importance of activities are also well‐documented. Females select social and skill‐based activities more often, while males lean toward active play and sports (Brown et al. 2011; Engel‐Yeger and Jarus 2008; Peral‐Suárez et al. 2020). Cultural norms reinforce these patterns, though they vary by context. For example, soccer is not gendered in Norway (Resaland et al. 2019), while in South Africa, domestic responsibilities differ by sex; females are responsible for chores such as caring for younger children, cooking and cleaning, and males are tasked with tending crops and gathering wood (Moses 2008; Visagie et al. 2017).

Despite this growing body of research, few studies have directly asked children which motor activities they consider important and wish to improve, especially across diverse cultural contexts and in relation to motor competence. This study addresses this gap by using a child‐friendly questionnaire with pictures to capture children's perspectives. By foregrounding children's lived experience, it contributes to a more inclusive and participatory model of paediatric rehabilitation. Specifically, we aim to examine which activities children perceive as important, and whether these perceptions vary by sex, cultural context and motor skill level. These insights can inform personalised, culturally relevant physical activity interventions that reflect children's real‐world needs and aspirations.

## 2 Methods

### 2.1 Participants

A cross‐sectional study was conducted with 1438 children from South Africa and Ghana. Participation in both countries was voluntary. Only children whose parents or caregivers provided written informed consent and who themselves gave assent were included. To determine activity preferences, the importance component of the motor coordination questionnaire (MoCQ‐I) was administered to both cohorts.

Different sampling strategies were used in the two countries. In South Africa, 1032 children aged 6–13 years (53% females and 47% males) were recruited from five primary schools around Potchefstroom representing different school quintiles and tested during school hours. In Ghana, 406 primary school children (51% females and 49% males) aged 6–12 years were recruited from three mainstream primary schools in local communities in the Eastern (more rural) and Greater Accra regions (more urban), using convenience sampling.

In South Africa, motor competence was assessed using the Movement Assessment Battery for Children, Second Edition (MABC‐2) (Henderson et al. 2007). Children scoring below the 16th percentile were classified as having probable DCD (pDCD), while those at or above the 16th percentile were considered typically developing (TD). In Ghana, the Developmental Coordination Questionnaire (DCDQ) (Wilson et al. 2009) age‐appropriate cut‐off scores were used to categorize the children into TD and pDCD groups.

Eligibility criteria included the following: (1) age between 6 and 13 years, and (2) the ability to participate in age‐appropriate motor activities. Children with diagnosed neurological or developmental conditions such as cerebral palsy, intellectual disabilities or attention deficit hyperactivity disorder were excluded due to the potential impact on motor competence and the validity of standardized assessments (Fernandes et al. 2024; Mashaal 2024).

### 2.2 Instruments

#### 2.2.1 MABC‐2

The MABC‐2 is a standardized test battery developed to identify children aged 3–16 years with impaired motor function (Henderson et al. 2007). The test consists of three age bands, each comprising eight items. Each age band is divided into three categories: manual dexterity, aiming and catching and balance. All three age bands were used in the study. The raw scores are converted to standard scores and percentile ranks. A percentile rank of five or less indicates severe motor coordination difficulties, while a percentile of 16 or less indicates moderate motor coordination difficulties. The MABC‐2 has demonstrated strong reliability (0.88–0.99 for subtests and 0.97 for total score) and validity for identifying motor coordination difficulties (Griffiths et al. 2018) and is one of the recommended tools in the DCD guidelines (Blank et al. 2019).

#### 2.2.2 Developmental Coordination Questionnaire

The DCDQ'07 questionnaire is a parent/teacher‐report screening tool used to identify motor coordination difficulties in children aged 5–15 years. It consists of 15 items across three domains: control during movement, fine motor and handwriting and general coordination. Chronological age and point score are used to classify children as TD or pDCD using age‐appropriate cut‐off values. Cut‐off points shown to indicate signs of DCD were used in this study according to age: ≤ 46 points (5–7 years), ≤ 55 points (8–9 years) and ≤ 57 points (10–15 years). The DCDQ shows good psychometric properties (Cronbach alpha = 0.94; overall sensitivity = 85%; and overall specificity = 71%) (Wilson et al. 2009; Cairney et al. 2008).

#### 2.2.3 Motor Coordination Questionnaire (MoCQ)

The MoCQ is a child‐friendly instrument designed to assess children's activities of daily living (ADL). This study used a culturally adapted version of the MoCQ, originally developed in the Netherlands (Adams et al. 2017). The adaptation process included a rigorous multistage developmental process to ensure cultural relevance and conceptual alignment with children's live experience.

Phase 1: Structured photo interviews with children to identify relevant motor activities. The development process began with structured picture‐based interviews in which children described the motor activities they engage in, how competent they feel in these activities, the importance they or their parents place on them and which activities they wished to improve. Children also identified activities that were irrelevant or missing from the existing picture set. This phase ensured that the questionnaire reflected the everyday motor tasks that were meaningful and culturally embedded in the participating communities.

Phase 2: Input from children, parents and teachers. Insights from Phase 1 were used to create a draft questionnaire that children, parents and teachers reviewed. They rated each activity based on difficulty, relevance, clarity and perceived competence and provided feedback on any activities that should be added, removed or reworded. This triangulated feedback strengthened the content validity of the instrument and captured a broad perspective on how motor tasks are valued and understood across social contexts.

Phase 3: Creation of MoCQ items. Drawing on the combined input from children, parents and teachers, the research team developed 15 core motor skill items representing common, culturally relevant activities. An additional item was added to reflect indigenous games, resulting in 16 items in total. Each item was designed to capture both perceived motor competence and perceived importance, enabling assessment of children's cognitive–affective responses to everyday motor tasks.

Phase 4: Integration of pictorial representations for cultural applicability. To facilitate use across linguistically and culturally diverse populations, each MoCQ item was paired with clear, contextually relevant pictorial representations. Because the names and meanings of activities differ across cultures, the use of visual cues helped ensure comprehension, reduced language burden and allowed multilingual children to respond accurately.

The final MoCQ consists of 16 items across two sections. Section A examines perceived competency, with children rating their performance on a 5‐point Likert scale from I am *not good at all* to *super good*. Section B examines the perceived importance of motor activities with ratings from *not at all important* to *very important*. The questionnaire ended with an open question: ‘What other activities not mentioned in the questionnaire are important to you’. One examiner worked with one to four children guiding them through each question before they completed their responses. If English was not their first language, an interpreter was present to support accurate interpretation of the instructions. For this study, only part B on importance (MoCQ‐I) was used focusing on children's perception of importance.

### 2.3 Procedure

Ethical approval was obtained from the Health Research Ethics Review Committee of the North‐West University, the Ghana Health Service (NWU00491‐19‐A1; Ghana: GHS‐ERC 052/05/19). Permissions were secured from the Department of Education (South Africa), the heads of the education districts (Ghana) and the school principals. Parents provided written consent, and children gave assent. Assessments were completed at the children's schools during school hours by trained healthcare professionals (with a master's in occupational therapy and a master's or PhD in Kinderkinetics and a minimum of 3 years of experience working with children) following standardized protocols.

### 2.4 Data Analysis

Descriptive statistics (frequencies, means, medians, range and standard deviations) were used to describe the sample. Differences between the South African and Ghanaian groups, between sexes and between motor coordination groups (TD vs. pDCD) were analysed using Mann–Whitney *U* tests for the total scores and crosstabs for differences in the frequency of the answer options. Effect sizes for the differences in total scores were calculated using the formula by Fritz et al. (2012). Counts and frequencies were used to summarize the activities mentioned in the open‐ended responses that were originally not part of the questionnaire. Spearman correlations were calculated between the total score on the MoCQ‐I and the DCDQ or the MABC‐2 total scores.

## 3 Results

### 3.1 Demographics

Of the 1438 participants, 685 were males, and 753 were females. In the Ghanian group, 214 were classified as TD, while 192 children had motor difficulties (pDCD). In the South African group, 426 were TD children, and 546 were identified with motor difficulties. Overall, 46% of the sample was TD, and 54% had motor difficulties (Table 1).

**TABLE 1:** Demographics of participants in the study.

Variable	Total group *n* = 1438	South Africa *n* = 1032^a^	Ghana *n* = 406
Age (mean ± SD)	9.34 ± 1.63	9.35 ± 1.59	9.33 ± 1.74
Males, *n/N total* (%)	685/1438 (48%)	488/544 (47%)	197/209 (49%)
Typically developing, *n* (%)	660 (46%)	446 (44%)	214 (53%)
Motor difficulties, *n* (%)	771 (54%)	579 (56%)	192 (47%)

*Note:* *n* = number of participants; % = percentage.

^a^ Total MABC‐2 scores were missing for seven children.

### 3.2 Group Differences

Total MoCQ‐I scores differed significantly between countries (*z* = −3.752, *p* < 0.001, *d* = 0.235), sex (*z* = −2.144, *p* = 0.031, *d* = 0.134) and motor coordination groups (TD vs. pDCD; *p* < 0.001, *d* = 0.335). Because different tools were used to identify motor difficulties in Ghana and South Africa, the comparisons between motor coordination groups were also performed separately by country. Motor coordination group effects remained significant when children were classified using the DCDQ (*z* = −4.636, *p* < 0.001, *d* = 0.472) and when classified using the MABC‐2 (*z* = −5.404, *p* < 0.001, *d* = 0.289). The spearman correlation between MABC‐2 and total MoCQ‐I scores was r_s_ 0.113, *p* 0.001 and between DCDQ and Total MoCQ‐I was r_s_ 0.233, *p* 0.001.

### 3.3 Activity Importance Rating at Item Level

**FIGURE 1:**
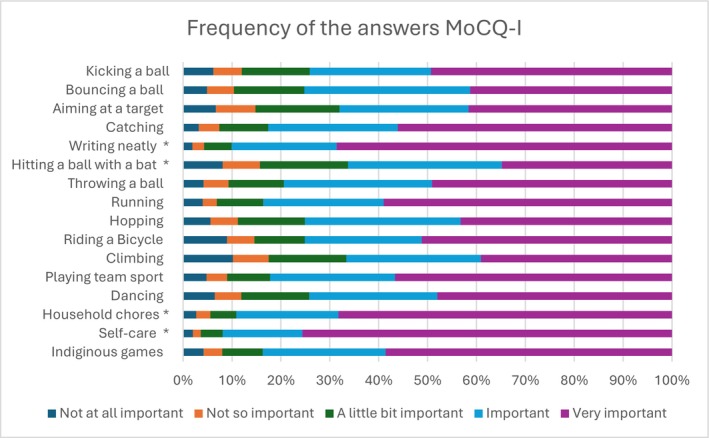
Frequency of the answers in the total group.

Figure 1 illustrates the frequency of the answers per question for the total group. The results indicate that household chores, self‐care and writing neatly are seen as the most important. Sixty‐eight percent of all children rated writing neatly as very important with only 1.9% reporting it as not important at all. Similarly, 68.3% of children rated household chores as very important. while 2.7% of children rated it as not important at all. Self‐care had the highest percentage of children rating it as very important with 75.6% of the total group, while only 2% rated it as not important at all. The results also indicate that 31.4% and 34.8% of children listed hitting a ball as important and very important, respectively.

**FIGURE 2:**
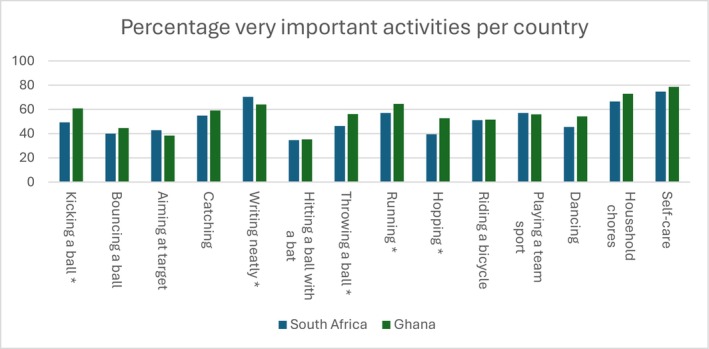
Percentage of activities rated as very important per country. *Significant differences in frequency between groups, *p* < 0.05.

Differences between countries: Figure 2 shows which activities children rated as very important per country. Children in both countries rated self‐care, writing neatly and household chores as very important. Similar frequencies between countries were found for bouncing a ball, aiming at a target, catching, hitting a ball, riding a bicycle, playing team sports, household chores and self‐care. However, kicking a ball was found to be more important in Ghana (60.8%) than in South Africa (49.3%). Similar differences were found for hopping (52.7%), running (64.5%), throwing (56.2%) and indigenous games (78.6%), which were scored higher by Ghanaian children compared to South African children (39.4%, 56.2%, 46.3% and 50.7%, respectively). Only writing neatly was perceived as more important by the South African children (70.3% versus 64.0%).

**FIGURE 3:**
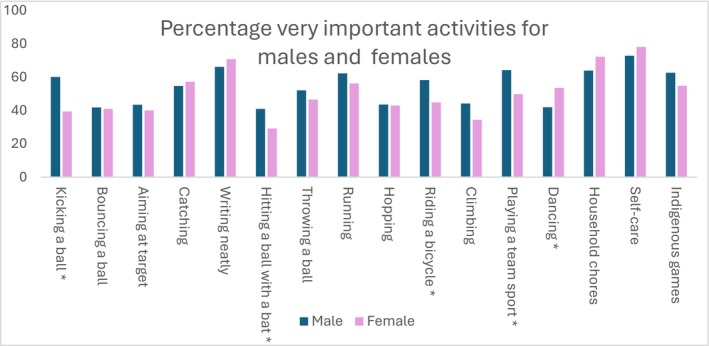
Percentage of activities rated as very important by sex. *Significant differences in frequency between groups, *p* < 0.05.

Differences between sexes: In Figure 3, the sex‐based differences can be seen. According to the results, males and females rated most activities similarly. More males than females indicated kicking a ball as very important (60.1% vs. 39.4%). Similar results were found for playing a team sport (63.7% vs. 50.9%) and riding a bicycle (58.2% vs. 44.8%). In contrast, females rated dancing higher than males did (53.5% vs. 41.9%).

**FIGURE 4:**
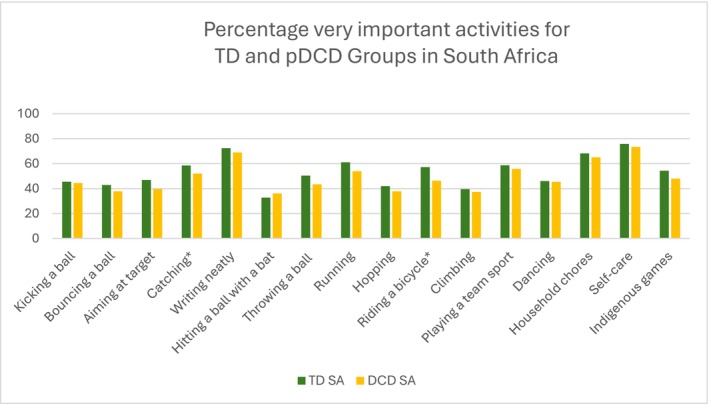
Percentage of activities rated as very important by motor coordination group in South Africa. *Significant differences in frequency between groups, *p* < 0.05.

**FIGURE 5:**
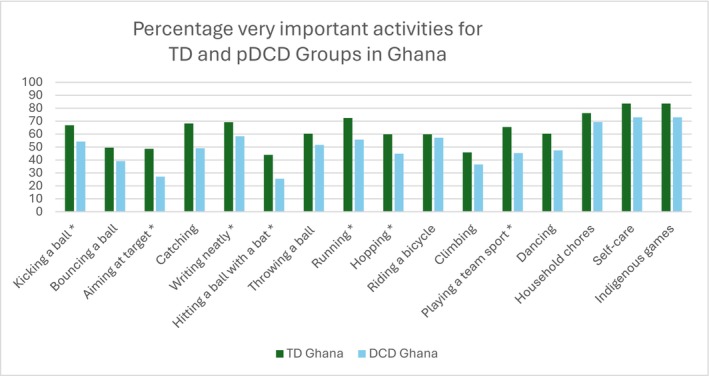
Percentage of activities rated as very important by motor coordination group in Ghana. *Significant differences in frequency between groups, *p* < 0.05.

Differences between TD and pDCD groups: The results indicate that many activities had similar frequencies independent of motor competency level and country (bouncing a ball, throwing a ball, climbing, dancing, household chores, self‐care and indigenous games). However, other activities were perceived differently in the two countries by children with and without motor problems (Figures 4 and 5). Differences in perceived importance between TD and pDCD groups were more frequent and larger in the Ghanian children (kicking a ball, aiming at a target, writing neatly, hitting a ball with a bat, running, hopping and playing team sport; all *p* < 0.05, Table 2).

**TABLE 2:** Overview of the cross‐tabulation results at item level for the different group comparisons.

Items	Countries	Sex	Ghana	South Africa
	Ghana vs. South Africa	Males vs. females	TD vs. pDCD	TD vs. pDCD
Kicking a ball	0.001 #	0.001 @	0.03	0.43
Bouncing a ball	0.091	0.123	0.07	0.35
Aiming at a target	0.155	0.046 @	0.001	0.19
Catching	0.119	0.320	0.001	0.044
Writing neatly	0.001 *	0.357	0.026	0.30
Hitting a ball with a bat	0.579	0.001 @	0.002	0.112
Throwing a ball	0.008 #	0.125	0.34	0.14
Running	0.008 #	0.143	0.01	0.19
Hopping	0.001 #	0.08 &	0.02	0.45
Riding a bicycle	0.153	0.001 @	0.003	0.004
Climbing	0.039	0.003 @	0.19	0.29
Playing team sport	0.055	0.001 @	0.001	0.48
Dancing	0.041	0.001 &	0.14	0.76
Household chores	0.065	0.007 &	0.22	0.75
Self‐care	0.37	0.17	0.12	0.85
Indigenous games	0.001 #	0.022 @	0.12	0.16

*Note:* # indicates higher ratings Ghana, * indicates higher ratings South Africa, @ indicates higher ratings males and & indicates higher ratings females.

**FIGURE 6:**
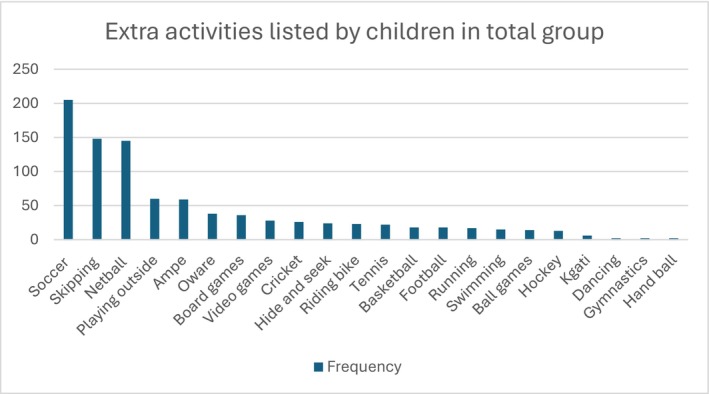
Frequency of activities mentioned as important but not listed in the questionnaire by the children in both countries.

Open question: When looking at the answers to the open question ‘What other activities not mentioned in the questionnaire are important to you’ (Figure 6), soccer, skipping and netball were the most listed activities with soccer chosen by 22% of children and skipping and netball by 16% of children, respectively. In addition, children listed playing outside (6%) and traditional games such as *Ampe* (6%) and *Oware* (4%) as activities they would like to participate in.

## 4 Discussion

This study investigated the activities children perceive as important and examined variations based on country, sex and motor coordination level. While children in South Africa and Ghana similarly rated self‐care, writing neatly and completing household chores as very important, notable differences in the frequencies of the answers emerged across countries, sex and motor coordination levels.

Ghana and South Africa present distinct cultural and environmental contexts that shape children's opportunities for movement. In Ghana, informal outdoor play is common, and culturally valued activities such as soccer and traditional games like *Ampe*, a rhythmic game involving jumping and clapping, are integral to childhood experiences (Ocansey et al. 2016). Soccer holds significant cultural importance and is widely played in community spaces. In contrast, South African children from middle‐ and low‐SES communities often have limited access to formal recreational facilities, with play occurring in informal or shared outdoor areas (Gerber et al. 2021). Safety concerns in some neighbourhoods may restrict outdoor play, influencing which activities children perceive as feasible or important. South Africa also has a strong tradition of organized sports such as rugby, netball and cricket, which may shape children's aspirations and preferences. These contextual differences help explain the patterns observed in the communities included in this study. Ghanaian children were more likely than their South African peers to rate kicking a ball and hopping as very important, reflecting the prominence of soccer and traditional games in Ghanaian culture. Similarly, Moses (2008) noted that South African children frequently contribute to domestic responsibilities, consistent with our finding that household chores were commonly rated as very important in both countries. Recognizing these cultural and environmental influences are essential for interpreting cross‐country variations in children's perceptions of meaningful motor activities.

Sex‐based differences were also observed. Males and females rated most activities similarly, yet males were more likely to value kicking a ball, riding a bicycle and playing team sports, whereas females rated dancing more highly. This corresponds with previous studies reporting gendered participation in sports, dancing and rhythmic gymnastics (Cherney and London 2006; Eyler et al. 2006; Peral‐Suárez et al. 2020; Resaland et al. 2019). Hendricks et al. (2019) reported that fewer South African females aged 9–11 participated in team sports compared to males, and Mayeza (2016) highlighted that soccer is culturally framed as a male activity, while netball is often reserved for females.

Differences were also observed between TD children and those with probable developmental coordination disorder (pDCD). Overall, children with pDCD rated the importance of motor activities lower, consistent with evidence that children with DCD may avoid activities that expose their motor difficulties, especially in social contexts (Engel‐Yeger and Kasis 2010; Mimouni‐Bloch et al. 2016). Increased effort required for motor planning and execution may lead to frustration, reduced self‐efficacy and eventual withdrawal from challenging motor tasks and also rating them as less important (Nobre et al. 2019).

The more detailed analysis at item level revealed that TD children and children with pDCD assigned similar importance to some activities such as bouncing a ball, throwing a ball, dancing, household chores, self‐care and indigenous games. However, other activities were rated differently for TD and pDCD, with more pronounced disparities in the Ghanaian group. The Ghanaian children with pDCD perceived many activities such as kicking a ball, aiming at a target, writing neatly, hitting a ball with a bat, running, hopping and playing a team sport as less important. One possible explanation is that the DCDQ, which includes a broad range of functional motor tasks (Wilson et al. 2009), may have a stronger conceptual overlap with the MoCQ‐I than the MABC‐2. However, the observed associations between the tools were low. Although both the MABC‐2 and the MoCQ‐I are related to motor competence, they differ in focus and measurement approach. The MABC‐2 is a performance‐based test emphasizing fine and gross motor skills under standardized conditions (Henderson et al. 2007), whereas the MoCQ‐I captures children's perceived importance of motor activities in daily life. These constructs are related but not equivalent: Performance tests measure actual ability, while perception‐based tools reflect subjective value and contextual relevance. Consequently, a child may rate an activity as highly important despite struggling to perform it, or conversely, may perform well on a task without considering it meaningful. This conceptual divergence, combined with cultural influences on perceived importance, helps explain the weak correlation between the used instruments.

Across both countries, soccer, skipping and netball emerged as the most frequently mentioned important extra activities. Including these examples in the questionnaire could reduce responses to the open‐ended question about unlisted important activities. These physically demanding activities require anaerobic and aerobic capacity, motor coordination and teamwork. Soccer and netball, for instance, involve short bursts of energy to intercept the ball (Davidson and Trewartha 2008; Reilly et al. 2000). Netball is particularly popular among females in South Africa, while soccer is widely played in both Ghana and South Africa (Mayeza 2016). Their popularity reflects not only children's interests but also the accessibility and cultural relevance of these games.

Traditional games such as *Ampe* and *Oware* were also mentioned by Ghanaian children, demonstrating the value placed on indigenous play. *Ampe* is a rhythmic game involving clapping and jumping with alternating feet in a group, where children compete and rotate turns (Ofori 2023). *Oware*, one of Ghana's oldest board games, involves placing pebbles in shallow pits and requires cognitive strategy, turn‐taking and fine motor skills (Ofori 2023). These culturally meaningful activities emphasize the importance of recognizing diverse forms of play and movement in child development, especially in non‐Western contexts where structured recreational programmes may be less accessible. This highlights the role of culture in shaping children's activity choices (Cherney and London 2006; Eyler et al. 2006; Resaland et al. 2019).

Overall, these findings affirm that children's perceptions of important activities are shaped by sociocultural context, gendered expectations and motor ability. This study underscores the value of directly asking children what they find important, rather than relying solely on observed participation or adult assumptions. For therapists and professionals in child development, these results emphasize the need for culturally responsive, child‐centred approaches that honour children's voices and create supportive environments for participation.

## 5 Limitations

This study has several limitations. First, different tools were used to assess motor coordination in the two cohorts: the MABC‐2 in South Africa and the DCDQ in Ghana. These instruments differ in format, scoring and focus (performance‐based vs. parent‐reported), which may have influenced the classification and prevalence rates of probable DCD across countries. Our cross‐country differences in motor competence may therefore reflect the parental perception of the children's motor abilities, rather than their actual motor skill level. Second, although the Ghanaian sample was substantial, it was smaller than the South African sample, potentially limiting statistical power and increasing the risk of sample bias. Lastly, no information about possible school‐based differences between the countries was available. Despite these limitations, the study contributes valuable insights into children's activity preferences and highlights important implications for child‐centred intervention planning across diverse settings.

## 6 Conclusion

This study explored children's perceptions of important activities across two countries, examining variations by motor coordination level and sex. While children in both Ghana and South Africa rated self‐care, writing neatly and household chores as highly important, several cultural and contextual differences influenced the prioritization of other activities. Children with pDCD may be less likely to rate motor activities as important, possibly due to the challenges these activities present.

These findings emphasize the importance of incorporating children's voices into activity planning, particularly in culturally diverse and low‐resource settings. Understanding what children value, not just what they do, can support the design of more engaging, meaningful and contextually relevant activities. Clinicians and professionals (coaches and PE teachers) should actively involve children in identifying meaningful goals and recognize how cultural norms, gender expectations and motor competence shape their priorities. In low‐resource settings, traditional and unstructured play activities such as *Ampe* or household chores can be harnessed as valuable therapeutic tools.

## Author Contributions

**Marisja Denysschen:** investigation, visualization, data curation, validation, writing – original draft, local project administration. **Rosemary Xorlanyo Doe‐Asinyo:** investigation, validation, writing – original draft, local project administration. **Dané Coetzee:** investigation, supervision South Africa, project administration, writing – review and editing. **Wilmarié du Plessis:** investigation, supervision South Africa, local project administration, writing – review and editing. **Emmanuel Bonney:** supervision Ghana, project administration, writing – review and editing. **Bouwien Smits‐Engelsman:** conceptualization, methodology, data curation, formal analysis, writing – review and editing.

## Funding

The authors received no specific funding for this work.

## Ethics Statement

Ethical approval was obtained in Ghana and South Africa before the data were collected.

## Consent

Parental consent was acquired prior to the data collection, and participants also gave assent to participate.

## Conflicts of Interest

The authors declare no conflicts of interest.

## Data Availability

Data are available on request due to privacy/ethical restrictions.
